# Reinstating verbal memories with virtual contexts: Myth or reality?

**DOI:** 10.1371/journal.pone.0214540

**Published:** 2019-03-29

**Authors:** Michel Juhani Wälti, Daniel Graham Woolley, Nicole Wenderoth

**Affiliations:** 1 Department of Health Sciences and Technology, ETH Zurich, Zurich, Switzerland; 2 Neuroscience Center Zurich (ZNZ), University and ETH Zurich, Zurich, Switzerland; University of Sydney, AUSTRALIA

## Abstract

When learning new information, contextual information about the encoding situation is stored in addition to the focal memory content. Later, these strings of extra information can help retrieve the learned content as demonstrated by experiments where contextual cues from an encoding situation facilitate remembering and improve memory performance when reinstated during retrieval. This context-dependent memory effect has been investigated over the course of several decades and has been demonstrated with many different types of contexts. Based on this, the widely held belief is that context-dependent memory is a strong and robust effect, with transferable substance for everyday learning and potential clinical applications. Here we report the results of a multi-study design investigating the influence of reinstated visual contexts on memory performance. Data from 120 participants were included in three studies comprising a variety of visual cues. We show convincingly that even rich, salient and fully surrounding visual contexts provided by virtual reality are not sufficient to induce effects of context-dependency in a free recall memory task. We also investigated contextual modulation of oscillatory brain activity in order to test the effect of reinstated neural contexts, which failed to evoke a robust effect when re-tested in an internal conceptual replication study. Moreover, a Bayesian sequential statistical analysis revealed moderate to strong evidence against the hypothesis that reinstatement of visual contexts benefits free recall memory tasks indicating that effects are small and may not be suitable for transfer into everyday learning.

## Introduction

In his famous Madeleine Episode, Proust describes a sensation that the taste of a sweet tart arouses an incredibly vivid remembering of the past, awakening memories that have not come to mind for a long time [1]. This kind of memory reactivation spawned by environmental cues is called context-dependent memory, a phenomenon that has been studied in memory research for many decades (for a review: [2]). The influence of contextual changes on behavior was first demonstrated by Watson [3] who was able to disrupt spatial memory performance in rodents by altering the orientation of the maze between learning and retention. Subsequent research extended this finding to other contextual manipulations (e.g. illumination: [4], odors: [5]) and memory domains (e.g. contextual fear conditioning: [6, 7, 8]) and generally confirmed the influence of context reinstatement on behavior. Encouraged by positive findings in other species, experiments on human subjects were conducted but early studies struggled to transfer the effect of context-dependent memory to human behavior [9, 10] until Godden and Baddeley [11] demonstrated context-dependency of human recall performance in a seminal study. Their unusual experiment showed that learning lists of words either underwater or on dry land would lead to better recall performance when the retrieval process was performed in the same natural environment compared to a change in context. Nowadays, the Godden and Baddeley study is part of every textbook in psychology and with the increasing availability of technology that provides easy access to virtual reality visualizations, it is tempting to transfer the context-dependent memory effect to everyday learning scenarios and potential clinical applications. However, previous research has shown that this effect is highly variable across studies [12–16], which lead Smith [17] and Smith and Vela [2] to propose various methodological principles and frameworks for how contextual information affects recall. In particular, they hypothesized that the use of materials that are rich and salient in content are favorable for ensuring that manipulations of contextual features affect memory performance. However, a systematic examination of how visual virtual environments need to be designed to evoke context-dependent memory improvements is currently lacking.

Here we investigate three hypotheses regarding the visual properties of memory enhancing contextual cues. Although many studies have found that simple context manipulations of local visual features, such as background or font color [18–24] are sufficient to reveal context dependent memory improvements, others could only find such an effect on memory performance when screen contexts were perceptually rich and salient [18–21]. First, we replicate Isarida and Isarida [23] to test the idea that reinstating simple visual cues has an effect on recall performance (Study 1) and then investigate how much visual content is needed for contextual information to have a reliable effect on memory performance when reinstated (Study 2).

Second, we investigate the effectiveness of local versus global contexts. Local contexts surround only the to-be-learned stimulus and can change quickly, while global contexts include a wider range of the surrounding environment, often contain contextual cues in multiple modalities and change very slowly [24]. Previous studies examined the effect of radically different global environments but revealed mixed results with some reporting a positive effect of reinstatement on memory (e.g. [11, 25, 26]), but others failing to show such an effect (e.g. [12, 15, 16]). It has been argued that local contexts are advantageous because they change quickly and are more easily associated with the learning stimulus. Whereas a conscious association in a global environment might be less likely, thus reducing its influence on the memory process [24]. Today’s technological advances allow the experience of different global environments without the effort of physically changing locations and therefore provide the possibility to compare local and global contextual changes in a more controlled setting. Fully surrounding virtual environments presented via a head-mounted display (HMD) represent an economical and well controlled way to quickly switch between environments and provide a unique sense of presence as the user is fully immersed in different virtual worlds. Here we test the effect of reinstated local versus global contexts, by comparing the impact of a local on-screen context and a virtually presented and fully immersive environment on memory (Study 2 and 3).

Third, we investigate whether memory recall can be enhanced via neural reinstatement, which can be interpreted as internal context. Various studies [27–31] have shown that specific patterns of neural activation measured during an encoding event reoccur during successful retrieval. This principle has been tested by encoding words which are displayed together with one of two different flickering backgrounds, each oscillating at a specific frequency [28]. Exposure to visual flickering patterns produces so called steady-state visually evoked potentials in visual areas of the brain, which appear at the same frequency as the visual input and can be measured using electroencephalography (EEG). Intriguingly, during the subsequent recognition task which was performed with a neutral background, it was found that successful retrieval was accompanied by intrinsically evoked neural activity at the same oscillatory frequency as extrinsically evoked during encoding, suggesting that neural reinstatement supports memory recall. Using a more causal approach, a recent study reported improvements of memory performance when oscillatory brain activity was experimentally modulated by applying electrical alternating current stimulation such that a specific oscillatory frequency was entrained during the encoding of words and subsequently reinstated during retrieval [32]. Here we test whether modulating and reinstating oscillatory brain activity by visual flickering stimuli improves memory recall (Study 2 and 3).

## Materials and methods

### Participants

Preceding the design of the studies and recruitment of the participants, a sample size calculation was carried out. This estimation was based on the effect sizes derived from 21 context reinstatement experiments, which used recall as test type without promoting explicit associative processing, i.e. forming intentional mental connections between a learning item and the context it is presented in [2]. An average effect size of 0.46, paired t-test as statistical measurement, statistical power set at a value of 0.8, and a significance level of 0.05 revealed a minimum set of 39 participants per experiment. In order to fulfill this requirement, 40 participants were included in each of the experiments. A total of 126 healthy young volunteers signed an informed consent document and participated in three studies. Six participants were excluded from further analyses due to technical issues or because instructions were not followed accurately (e.g. looking away from screen or closing eyes during recall). Here we report a total of 120 participants (female: 60, mean age = 23.96, SD = 3.64). The study was approved by the local ethics committee (Ethics Committee Zurich) and all methods were performed in accordance with the committee’s guidelines and regulations.

### General design and procedure

**Fig 1:**
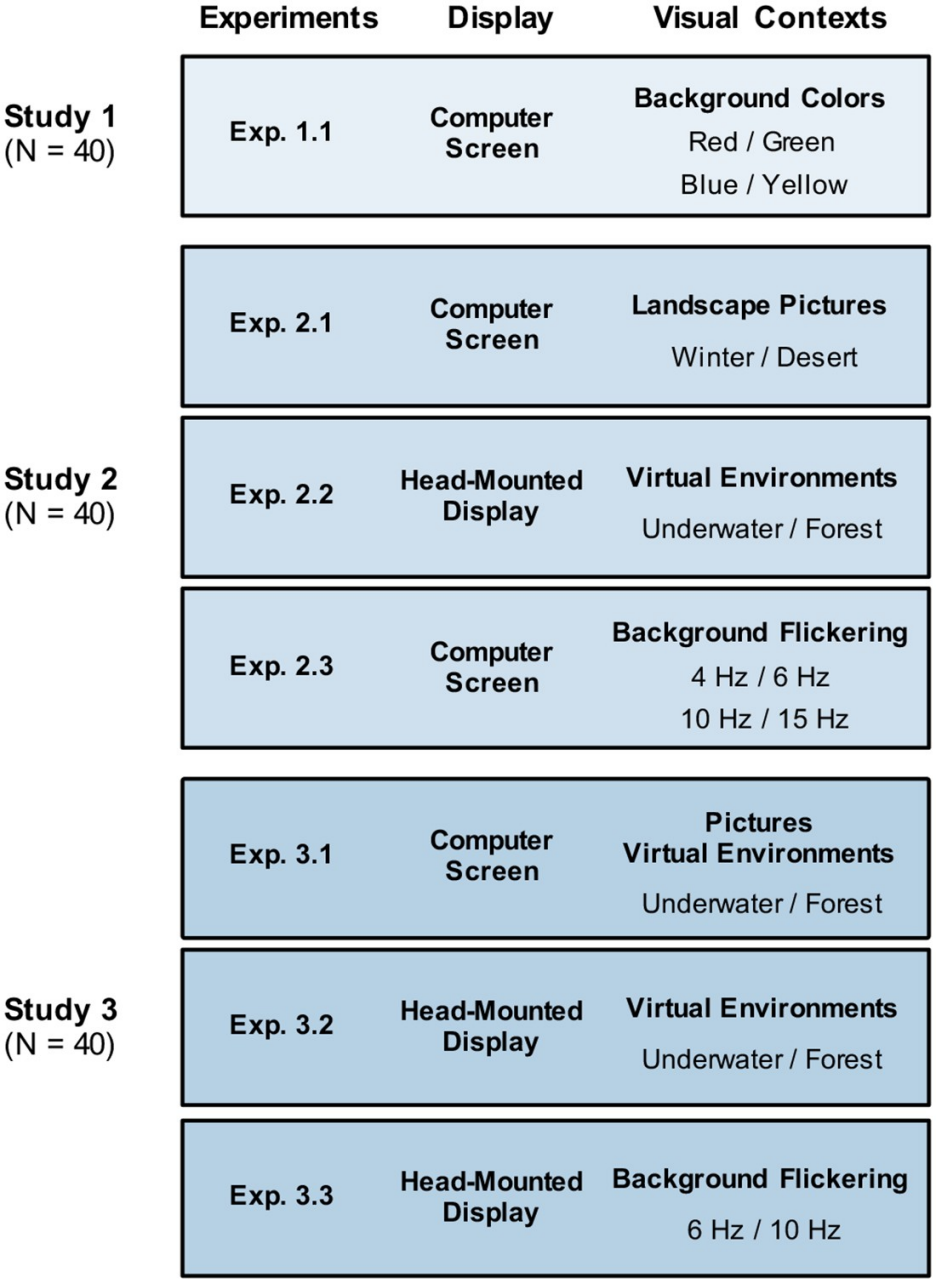
Multi-study design. Multi-study design consisting of three studies and seven experiments in total. In each experiment two visual contexts were presented during encoding, equally distributed across the learning items.

**Fig 2:**
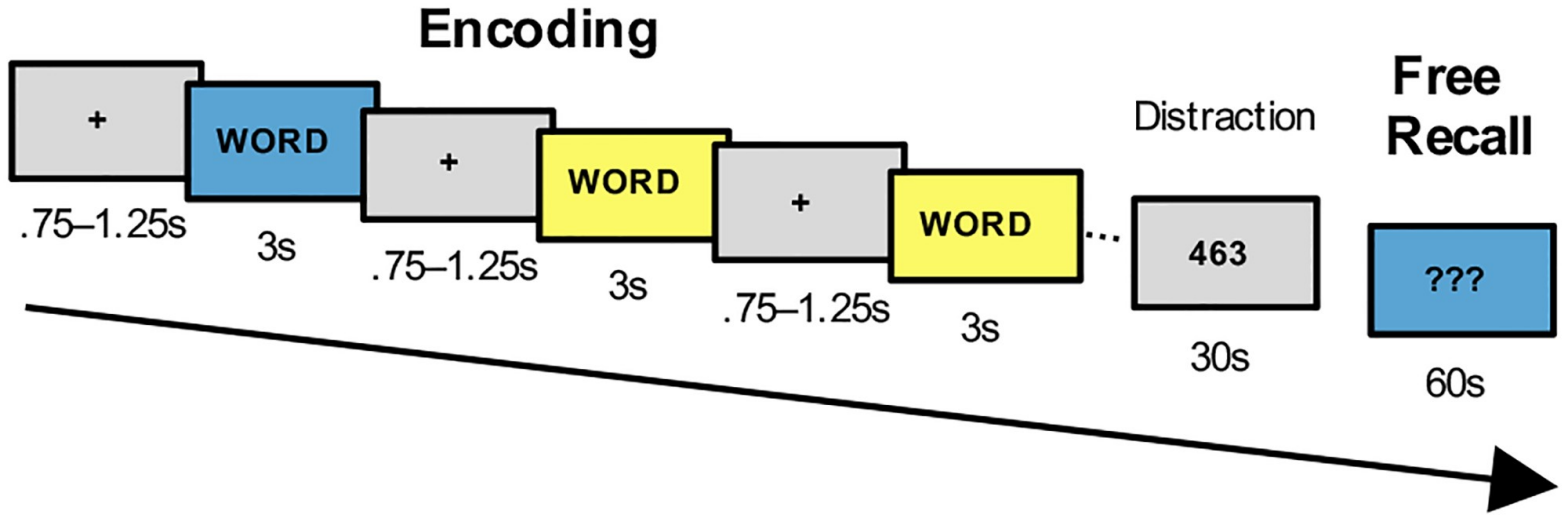
Experimental design. In a free recall memory task, contexts were visually presented in the background of 24 words. Subsequently, following a 30 second distractive counting task, participants were asked to freely recall all remembered words within 60 seconds, while only one of the encoding contexts was reinstated. Note that for Study 1, Pictures Landscape and Flickering Screens in Study 2 words were presented for 3 seconds at the same time as the contextual background was presented. Procedure for Virtual Environments in Study 2 and all three experiments in Study 3 differed in regard that words were presented 2 seconds after the contextual background appeared (and stayed in front of the background for 3 seconds).

We report three studies which consisted of maximally three experiments, each testing the effect of a specific visual context property on free recall memory tasks (Fig 1). Common to all experiments was that two contextual settings were visually presented as a background to 24 words that participants were instructed to memorize. The presentation order of the two contexts randomly changed word-by-word, with the restriction that no more than three successive contexts were the same and that both were presented with the same number of words, resulting in 12 words per context [23]. Subsequently, following a 30 second distractive counting task requiring participants to count backwards in steps of 7, 9 or 13, they were asked to freely recall all remembered words within 60 seconds, while only one of the encoding contexts was reinstated (Fig 2). This design was adopted from a previous study [23] and was chosen in order to replicate and build on findings using simple visual context modulations causing context-dependent effects on memory performance. The number of words per context (n = 12) was chosen according to previous studies showing that this number of items per context is small enough to avoid effects of cue overload [11, 17, 23]. Further principles of successfully modulating memory performance by reinstating contextual cues were taken into account, such as, (i) not encouraging subjects to self-generate context cues, (ii) use of recall instead of recognition tests, and (iii) encoding of each item in only one contextual setting to avoid decontextualization [2, 17].

### Word lists

In each of the experiments, participants were required to study 24 two-syllable German nouns in two different alternating contextual settings. A total of 72 nouns from the Berlin Affective Word List (BAWL-R) were split up into three word lists with the restriction of having an emotion-score between -0.6 and +0.6, an arousal-score of less than 3.0, and a frequency of 10 to 150 (per 1 Million). Alongside these objective criteria, each word was screened regarding its relatedness to other words from the same list and was, in cases of strong relationships, replaced. Also, words were excluded when obvious connections to one of the visual contexts could be identified. The three word lists were randomly used in one of three different experiments.

### Display of visual contexts

Visual contexts were displayed either on a computer screen (15.4-inch) or via a head-mounted display (HMD) to create fully surrounding virtual environments (Oculus Rift DK2, Oculus VR, LLC). In both cases participants were seated comfortably in a chair with an approximate distance of 60 cm to a computer screen. During the memory task (encoding–distraction task–recall) the light was switched off to reduce any visual influences other than what was displayed on the computer screen or via HMD. To create and display the visual contexts, as well as for recording of the verbal listing of remembered words, Unity 3d (Version 5.6, https://unity3d.com/) was used.

### Design and procedure of Study 1

Study 1 was set up as a replication of the Isarida and Isarida study (Experiment 1 in [23]) showing the effect of simple background color modulations on memory performance. A pair of background colors was randomly selected from two pairs (light red and light green, or light yellow and light blue), with the restriction that both color pairs were distributed equally across participants. Each word was presented for three seconds in black font in the center of a colored screen. A black fixation cross on a gray screen was presented between words for 0.75 to 1.25 seconds. During recall, auditory recording was carried out and later analyzed. Our design was a replication of Isarida and Isarida [23] except that we used German nouns instead of Japanese characters. Based on previous reviews and meta-analyses, a design that uses free recall to test memory is highly suitable to test contextual reinstatement on memory performance since it prevents decontextualization of learning items, avoids cue overload and does not encourage participants to self-generate context cues. Free recall data from 40 participants were included (female: 19, mean age = 23.3, SD = 3.27).

### Design and procedure of Study 2

Study 2 contained three experiments (2.1–2.3) with varying richness, saliency and immersiveness of the visually presented contexts (Fig 1). In comparison to the simple visual contexts in Study 1, in Experiment 2.1 we used more distinct and visually richer contexts, showing one of two landscape images (desert or winter landscape) presented alongside the to-be-learned words. With the intention of displaying fully surrounding visual contexts to expand the number of visual cues, and giving rise to a sense of immersion into the context, virtual environments were used in a second experiment. Inspired by the seminal study of Godden and Baddeley [11], Experiment 2.2 showed either an underwater scene or a scene on land (here: forest) which was displayed via a head-mounted display to the participants while to-be-remembered words were presented in the center of the visual field. The general procedure of experiment 2.2 was similar to experiment 2.1, however, the presentation of fully surrounding virtual environments via HMD, required two small adjustments: First, preceding the experiment, participants were familiarized with the environments by presenting both scenes, each for 30 seconds. Second, to avoid confounds that might have been caused by switching between the two virtual environments, an additional two seconds delay was added before each word appeared in the center of the visual field. The duration of word presentation was three seconds, as in the other experiments. Finally, in Experiment 2.3 we tested the concept that memory performance is affected by reinstating brain oscillations via flickering visual stimuli which were displayed on a computer screen. Therefore, words were presented in front of a gray and white flickering background. During encoding two flickering frequencies were randomly selected from two pairs (i.e. either 6 Hz and 15 Hz, or 4.29 Hz and 10 Hz) to induce steady-state responses at the respective frequency and its harmonics [33]. The choice of the frequencies was inspired by previous research, depicting reinstated steady-state responses during successful memory retrieval [28] and was constrained by the refresh rate of the computer screen used in the experiments (60 Hz). As in the other experiments, throughout recall, flickering in one of the encoding frequencies was displayed to reactivate oscillatory brain responses induced during encoding of half of the words. Research on steady-state visually evoked potentials has shown convincingly that the presentation of a flickering visual stimulus in the range of 1Hz to 100Hz produces a robust response in visual areas of the brain in the corresponding frequency (for reviews: [34, 35]). The general procedure of experiment 2.3 was identical to experiment 2.1. 40 participants (female: 22, mean age = 24.4, SD = 3.68) participated in the three experiments, which were carried out successively with a break of five minutes after the first and second experiments. Three different lists of words were used and each list was paired with one of the experiments. The order of the experiments, as well as the pairing of the word lists with Experiment 2.1–2.3 were counter-balanced across participants.

### Design and procedure of Study 3

Study 3 was carried out as a conceptual replication of Study 2 and to control for potential confounds which might have influenced overall memory performance. 40 participants (female: 19, mean age = 24.1, SD = 4.84) participated in three experiments 3.1–3.3. Similar to Study 2, Experiment 3.1 showed visual cues displayed on a computer screen which depicted a still scene either underwater or in a forest. This time, these scenes were part of the virtual environment used in Experiment 2.2 and 3.2 to test whether the immersiveness of the visual cue affects the context-dependent memory effect or general recall performance. Experiment 3.2 was an exact replication of Experiment 2.2. Finally, Experiment 3.3 was a conceptual replication of Experiment 2.3 and investigated the effects of oscillatory reinstatement on memory performance by using the same gray-white flicker stimuli as in Experiment 2.3, except this time they were presented across the full visual field via HMD. Importantly, all Experiments of study 3 followed the exact same timing of stimulus presentation, i.e. after presenting the visual context cue, there was a 2 second delay until the word was displayed.

### Data analysis and statistics

Data from all three studies were analyzed with a main focus on the comparison of recall performance of words with a reinstated context versus words that were presented without reinstatement of contextual cues during recall. Paired t-tests were used to detect differences between the amount of recalled words with reinstated context, versus the amount of recalled words with a change in context within each experiment. On account of our directional hypothesis of a positive effect of reinstatement on memory performance, one-tailed tests were used, with a significance level of p < 0.05. In addition, we analyzed general memory performance across the experiments that were performed by the same subjects (one-way repeated measures ANOVA’s and paired sample t-tests for post-hoc comparisons). Matlab (Version R2014b, https://www.mathworks.com) was used for initial analysis of word count, SPSS (Version 23, https://www.ibm.com) for statistical analyses. To extend interpretation of our results, Bayesian statistics were employed using JASP software (Version 0.8.6, https://jasp-stats.org/). These tests produce Bayes factors (BF10 and BF01), which are graded measurements indicating evidence in favor of the null (H0) or the alternative hypothesis (H1) [36]. Expressing evidence for or against hypotheses on a continuous scale (e.g. [37]) is one main advantage of Bayesian statistics over a frequentist approach (for a systematic review on Bayes analyses in psychology experiments see [38]) which allows no conclusion about the null hypothesis. Under the assumption that the null hypothesis is true, a p-value reflects the probability of obtaining an effect at least as extreme as the one in a tested data sample. Bayes statistics on the other hand allow a direct comparison between the null and the alternative hypothesis. For example, when the Bayes factor BF10 equals 5, the data are 5 times more likely under H1 than under H0. Additionally, the Bayesian approach allows the monitoring of the evidential trajectory as the data accumulate [39]. We used such sequential analyses to illustrate how the evidence of H1 (e.g. words with reinstated contexts are recalled better) over H0 (no difference of contextual reinstatement regarding memory performance) changed with an increasing number of subjects. Note that for this, we pooled data from conceptually similar experiments (e.g. Exp. 2.3 and Exp. 3.3, both with flickering backgrounds) in order to increase the number of data points and therefore statistical power. This approach entails a statistical inaccuracy, as we treat repeated measurements (from within experiments) as independent observations. For reasons of understanding and to simplify interpreting the size of Bayes factors, we used the suggested scale of Harold Jeffreys [37] (BF10 = 1: no evidence; BF10 = 1–3: anecdotal evidence for H1; BF10 = 3–10: moderate evidence for H1; BF10 = 10–30: strong evidence for H1; BF10 = 30–100: very strong evidence for H1 and the inverse cutoffs–i.e. 1/3, 1/10, 1/30, 1/100 –to describe the evidence in favor of the H0). Cauchy prior width was set to JASP’s default r = 0.707. The datasets generated and analyzed in the current study, are available at Open Science Framework (https://doi.org/10.17605/OSF.IO/C5369).

## Results

**Fig 3:**
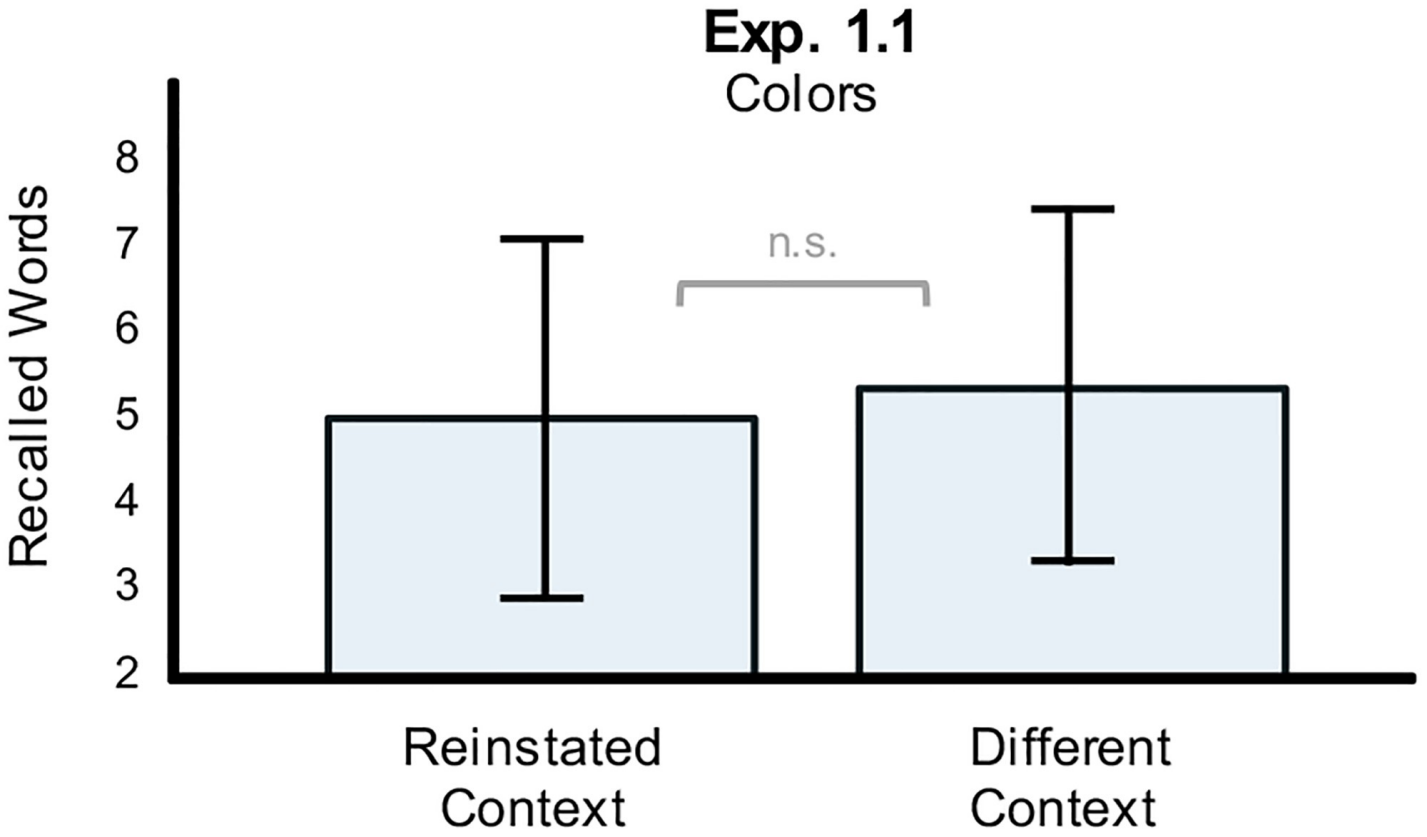
Memory performance in Study 1. Comparison of free recall performance for reinstated contexts and not-reinstated contexts in Study 1. Depicted are average recall performances and standard deviations across subjects. Background colors did not show a significant effect on memory when reinstated.

In order to replicate previous findings by Isarida and Isarida [23], our first study examined whether a background-color context effect would appear if two background colors were changed randomly word-by-word. Free recall data were analyzed and the number of correctly recalled words determined. Recalled words were classified as reinstated and not-reinstated according to whether the background color at test was the same or different as during encoding. From the retrieval of 12 possible reinstated words, an average of 5.1 were recalled (M = 5.1, SD = 2.1), which is less than the average of 5.5 recalled not-reinstated words (M = 5.5, SD = 2.1; Fig 3). A paired t-test revealed no significant difference between the recall performance for reinstated and not-reinstated words (t(39) = -1.292, p = 0.898, Cohen’s d = -0.204) and the obtained Bayes factors of BF10 = 0.1 and BF01 = 12.5 provide strong evidence in favor of the null hypothesis (H0), i.e. against the context-dependent memory hypothesis. Additionally, recall performance was slightly *worse* for the reinstated than the not-reinstated condition. Thus, we could not replicate Isarida and Isarida [23] and found no experimental support for a context dependent memory effect when using simple color context cues in Study 1.

**Fig 4:**
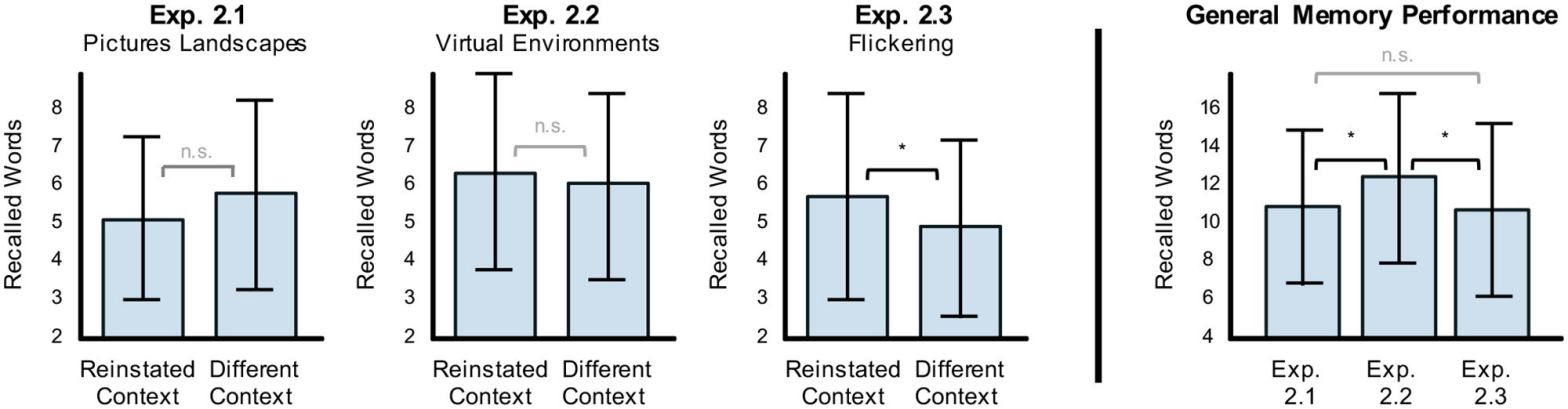
Memory performance in Study 2. Comparison of free recall performance for reinstated contexts and not-reinstated contexts in the three experiments of Study 2 (left side): Pictures of landscapes (Exp. 2.1; left panel) and virtual environments (Exp. 2.3; right panel) did not reveal an effect of reinstatement on memory performance. Reinstated flickering screen backgrounds, however, led to better recall (Exp. 2.2; middle panel). Comparison of general memory performance across the experiments within Study 2 (right side): In Study 2, the amount of recalled words in Virtual Environments was significantly higher compared to the other two experiments. Statistically significant effects are indicated by *: p < 0.05.

Next, we investigated how richer and more salient visual features affect the context dependent memory effect in Experiment 2.1–2.3. The analysis of free recall data shows that no significant context effect could be found when visually rich background pictures showing two different landscapes were used (Fig 4 left side, left panel; t(39) = -1.723, p = 0.954, Cohen’s d = -0.272) and that the number of remembered reinstated words (M = 5.2, SD = 2.2) is again slightly fewer compared to not-reinstated words (M = 5.9, SD = 2.5). Analysis of Bayes factors again revealed strong evidence for the null hypothesis, i.e. against the context-dependent memory hypothesis (BF10 = 0.1, BF01 = 14.8). Increasing visual richness and immersiveness of the environmental context by virtually presenting two different fully surrounding environments in Experiment 2.2 revealed that the number of recalled reinstated words (M = 6.5, SD = 2.6) was slightly higher than the recalled not-reinstated words (M = 6.1, SD = 2.5; Fig 4 left side, middle panel). However, t-statistics revealed no significant context effect (t(39) = 0.934, p = 0.178, Cohen’s d = 0.148), and Bayes factors were also small (BF10 = 0.4, BF01 = 2.4) revealing anecdotal support for the null hypothesis, i.e. against the context-dependent memory hypothesis. In Experiment 2.3, the screen background provided the contextual cue by flickering at one of two frequencies during encoding and recall. Because a test of normality (Shapiro-Wilk) revealed that the data were not normally distributed, a Wilcoxon signed-rank test was used to compare the two paired measurements. This test showed that reinstatement of steady-state visually evoked potentials at a specific frequency improves recall performance (M = 5.8, SD = 2.8) compared to not reinstated conditions (M = 5.0, SD = 2.4) (W = 458.500, p = 0.023, matched rank biserial correlation (mrbc) as measure of effect size = 0.118; Fig 4 left side, right panel). In addition, Bayes factors indicate that there is anecdotal evidence in favor of the alternative hypothesis H1 (BF10 = 2.8, BF01 = 0.4), i.e. this is the first evidence revealed by our studies weakly supporting the context-dependent memory hypothesis. Further analyses indicated that no frequency-specific effect on memory performance could be found. An ANOVA with recall context as a fixed factor and total recall performance as a dependent variable revealed no significant difference between the four flickering frequencies (4.29 Hz, 6 Hz, 10 Hz, 15 Hz) presented during recall (F(3,36) = 0.303, p = 0.823). Similarly, comparing the effect of different flickering frequencies during encoding on memory performance, independently of whether they were reinstated during recall, we found no difference between the four frequencies (F(3,76) = 0.420, p = 0.739).

General memory performance differed across the three experiments from Study 2 (ANOVA, F(2,78) = 6.057, p = 0.004; Fig 4 right side), with significantly better memory performance for Virtual Environments than Landscape Pictures (post-hoc Bonferroni t(39) = -2.793, p = 0.024, Cohen’s d = -0.442), or the Flickering Screens (post-hoc Bonferroni t(39) = 2.982, p = 0.015, Cohen’s d = 0.472). No significant difference between Landscape Pictures and Flickering Screens was found (t(39) = 0.452, p = 1.000, Cohen’s d = -0.071). Thus, Study 2 revealed two interesting results: first, visually entraining a specific frequency via a flickering background might mediate a small contextual memory effect in agreement with previous reports [28, 32]. Second, overall memory performance was significantly better when stimuli were encoded within a virtual environment than with other context cues. However, both results need to be interpreted with care. First, the statistical evidence in support for context dependent memory improvements induced by visual entrainment (Experiment 2.3) was weak (according to Jeffreys’ scale “anecdotal”), requiring an internal replication to rule out a random result (particularly, since three out of four experiments revealed anecdotal to strong support against contextual memory effects). Second, the superior general memory performance when words were encoded within a virtual reality environment (Experiment 2.2) might have resulted from a slightly longer inter-stimulus interval between the presentation of words in the Virtual Environments (i.e. additional two seconds compared to the other experiments) since participants experienced more time for the encoding of each word.

**Fig 5:**
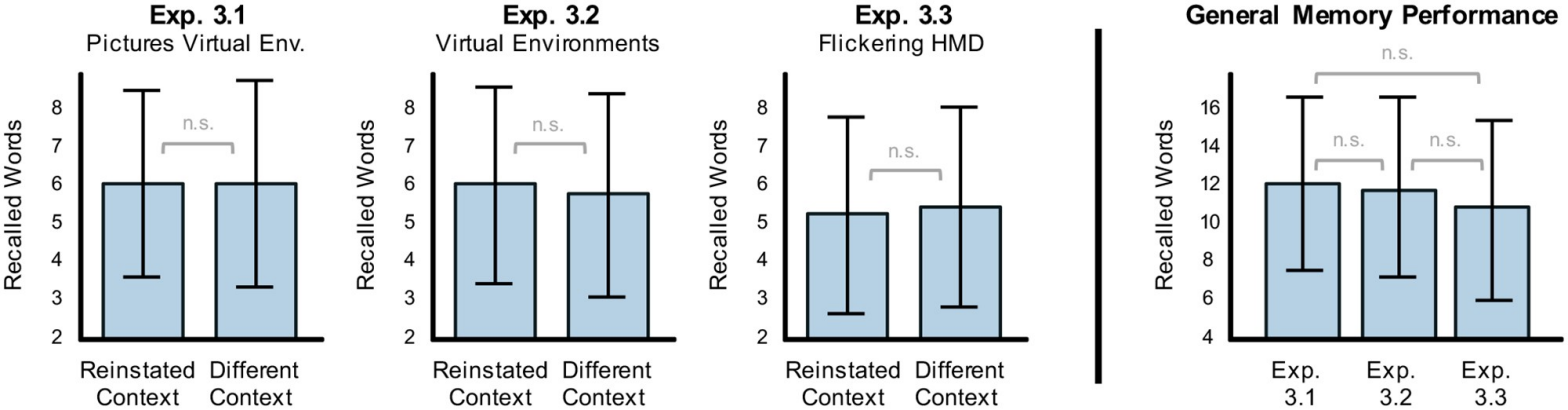
Memory performance in Study 3. Comparison of free recall performance for reinstated contexts and not-reinstated contexts in the three experiments of Study 3 (left side): Screen pictures of the virtual environments (Exp. 3.1; left panel), virtual environments (Exp. 3.2; middle panel), and also flickering backgrounds presented via HMD (Exp. 3.3; right panel) did not reveal an effect of reinstatement on memory performance. Comparison of general memory performance across the experiments within Study 3 (right side): No difference between the three experiments was observed.

Therefore, we performed Study 3 which was a conceptual replication of Study 2 but addressed this potential confound by using the exact same timing across all three experiments 3.1–3.3. Replicating the results from Study 2 using static landscapes (Experiment 3.1, Fig 5 left side, left panel) revealed no significant difference between the reinstated (M = 6.2, SD = 2.5) versus different context conditions (M = 6.1, SD = 2.7) (t(39) = 0.128, p = 0.449, Cohen’s d = 0.020). Moderate evidence in favor of H0, i.e. against the context-dependent memory hypothesis, was provided by the resulting Bayes Factors (BF10 = 0.2, BF01 = 5.3). Also, using virtual environments (Experiment 3.2, Fig 5 left side, middle panel) did not show a significant effect of context reinstatement on memory performance (Wilcoxon signed-rank test: W = 347.500, p = 0.196, mrbc = -0.152; BF10 = 0.436, BF01 = 2.296), even though slightly more words were remembered in the reinstated (M = 6.2, SD = 2.6) than in the non-reinstated condition (M = 5.9, SD = 2.7). Since Experiment 3.2 was an exact replication of 2.2, we pooled the data of 80 participants but still found no significant effect of context reinstatement using virtual environments on recall performance (reinstated context: M = 6.3, SD = 2.6 vs. different context M = 6.0, SD = 2.6; Wilcoxon signed-rank test: (W = 1281.500, p = 0.128, mrbc = -0.209). Resulting Bayes Factors (BF10 = 0.5, BF01 = 1.8) reveal anecdotal evidence in favor of H0, i.e. against H1.

Experiment 3.3 was a conceptual replication of 2.3 (methodological differences were the use of the HMD display instead of a computer screen and a 2 second longer inter-stimulus interval), however, this time reinstatement of oscillatory activity did not significantly affect memory performance (t(39) = -0.753, p = 0.772, Cohen’s d = -0.119; Fig 5 left side, right panel). In fact, recall in the reinstated condition was slightly worse (M = 5.3, SD = 2.6) than in the non-reinstated condition (M = 5.6, SD = 2.6) and Bayes Factors (BF10 = 0.1, BF01 = 9.6) revealed moderate evidence in favor of H0. Finally, we compared general memory performance across all three experiments from Study 3 (Fig 5 right side). In contrast to Study 2, there was no significant difference between experiments 3.1–3.3 even though there was a trend towards significance most likely driven by better recall using virtual reality pictures than the flickering screen condition (F(2, 78) = 2.564, p = 0.083). Note, however, that we found no support for the finding from Study 2 that general memory performance is better in fully surrounding virtual environments.

**Fig 6:**
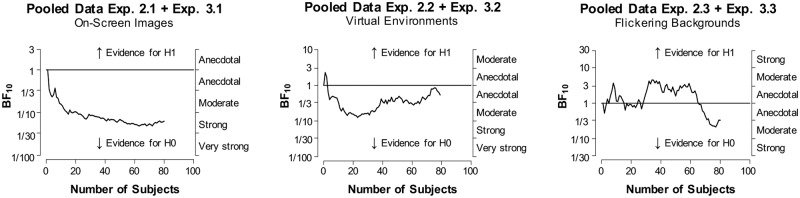
Sequential analysis of pooled data. Graphical output for the sequential analysis of pooled data for Studies 2 and 3. Displayed is the flow of evidence for H1 over H0 as the data (number of subjects) accumulate. Left panel: pooled data of Exp. 2.1 and 3.1, middle panel: pooled data of Exp. 2.2 and 3.2, right panel: pooled data of Exp. 2.3 and 3.3.

In order to further investigate our data and to depict the consistency of our findings, in an additional analysis step we pooled results from conceptually similar experiments across Study 2 and 3 (Experiment 2.1 together with Experiment 3.1, and Experiment 2.3 together with Experiment 3.3). Pooled data from experiments using background screen images (Experiment 2.1 and Experiment 3.1) revealed no significant difference between reinstated (M = 5.7, SD = 2.4) and not-reinstated conditions (M = 6.0, SD = 2.6) (t(79) = -1.075, p = 0.857, Cohen’s d = -0.120). Further, Bayes Factors (BF10 = 0.1, BF01 = 15.9) reveal strong evidence in favor of the null hypothesis (H0), i.e. against the context-dependent memory hypothesis. Similarly, pooled data from Experiments 2.3 and 3.3 (flickering backgrounds) revealed no significant difference between reinstated and not-reinstated conditions (Wilcoxon signed-rank test: W = 1306.500, p = 0.205, mrbc = -0.194) and moderate evidence in favor of H0, i.e. against the context-dependent memory hypothesis, can be concluded from the Bayes Factors (BF10 = 0.3, BF01 = 3.0) which was derived from Bayesian updating after 80 participants. Fig 6 depicts graphical outputs from the sequential analyses of the three pooled datasets. While the reinstatement of simple background images on a computer screen reveal increasing evidence for the null hypothesis with increasing number of subjects (Fig 6 left panel), trajectories of the other two pooled datasets show no such clear direction. Evidence for H0 in the two experiments using Virtual Environments (Exp. 2.2 and 3.2) decreases from moderate to anecdotal with increasing number of subjects (Fig 6 middle panel). On the other hand, the flickering experiments (Exp. 2.3 and 3.3) reveal more evidence for H0 over H1 only after approximately 60 subjects, increasing afterwards to moderate-anecdotal evidence in favor of H0 (Fig 6 right panel).

Potential order effects of the experiments, as well as putative differences between the chosen word lists, were tested by additional between-group ANOVA’s on the data from Study 2 and Study 3. We found no significant effect of experimental order (Study 2: F(2, 117) = 0.016, p = 0.984; Study 3: F(2, 117) = 0.084, p = 0.919), or a significant effect of word list on memory performance (word lists Study 2: F(2, 78) = 1.519, p = 0.225; word lists Study 3: F(2, 78) = 0.246, p = 0.782). Because participants in Study 2 and 3 completed the three experiments successively, one concern might be that they could determine the procedure and, arguably, the purpose of the experiments. As a result, participants may have learned that the visual contexts only serve as a memory aid for some of the words during recall. Thus, they could have adapted their strategy by deliberately ignoring the contexts during encoding after the first experiment. Consequently, the experiments that were completed second and third, in contrast to the first experiment, would not show an effect of context reinstatement on memory. However, dividing the analysis for both Studies 2 and 3 regarding the order of the experiments into first, second and third, revealed that none of the experimental order positions, revealed a significant effect of context reinstatement on memory performance (see Supporting Information, S1 Table). Note that we cannot rule out the possibility of participants anticipating the retrieval design in the first experiments of Study 2 and 3.

A recent study on free recall experiments divided the retrieval process in to two stages [40]: In a first stage, people tend to empty their working memory storage. This stage is affected by the serial-positions of the encoding items (recency effect and primacy effect). The second stage is more independent of working memory processes and involves searching for memorized information. To test, whether effects of context reinstatement would more likely occur in one of the two stages, individual recall data from all seven experiments were divided in half regarding the order of recall (first half of recalled words and second half of recalled words) resulting in 14 sets of data. Two-sided paired t-tests were used to compare reinstated and non-reinstated words regarding recall performance in each of the data sets (see Supporting Information, S2 Table). None of the comparisons revealed a significant difference. Further analysis of data pooled across all 7 experiments revealed no significant difference on memory performance between reinstated and different contexts either in the first half (t(279) = 1.170, p = 0.243, Cohen’s d = 0.095) or second half (t(279) = -0.273, p = 0.785, Cohen’s d = 0.023) of the recalled words. Our results suggest that neither the first or second stage of recall was affected by reinstated visual contexts.

In a final statistical analysis, we conducted a one-way ANOVA with random effect for study number and found no difference in general memory performance across the seven experiments, despite their pronounced differences in visual richness and immersiveness (F(6, 273) = 1.298, p = 0.258; note that the statistical power of this analysis was somewhat inflated since we treated data from the same subject as independent measurements).

## Discussion

In the present study, we investigated how visual virtual environments need to be designed to evoke context-dependent memory improvements. In particular, we tested the influence of the richness of the visual cue, local versus global features of the visual context and neural reinstatement via flickering backgrounds on memory recall under reinstated and not-reinstated conditions. Overall, 6 out of 7 experiments revealed anecdotal to strong evidence in favor of the null hypothesis, even when pooling data across conceptually similar experiments to perform a well-powered statistical analysis. Only one experiment, which used a local flickering cue with the aim of employing a neural reinstatement mechanism, resulted in significantly better recall of words in a reinstated versus non-reinstated context based on a Wilcoxon signed-rank test (p = 0.023). However, Bayesian statistics yielded only anecdotal evidence in favor of a context-dependent memory advantage and a conceptual replication using global flickering cues provided via the HMD and slightly longer encoding times failed to replicate the initial finding. Thus, overall, the most parsimonious interpretation of our series of studies is that visual cues, including immersive virtual environments, evoke only small to no contextual memory effects. Moreover, our data suggest that overall memory performance depends significantly on encoding time but not on the visual context.

### General methodological considerations

It has been argued previously that the chosen study design might have an important influence on whether or not context-dependent memory effects can be experimentally demonstrated. The design of our studies replicated earlier approaches to investigate context-dependent memory (e.g. [23, 41]) and followed several guidelines regarding methodological aspects which would influence the effectiveness of reinstated contexts as identified in previous reviews and meta-analyses [2, 42]. These guidelines recommend (i) the use of free recall tasks rather than recognition tasks, (ii) the monitoring of cue overload, (iii) avoiding repetition of stimulus presentation, and (iv) limiting stimuli difficulty [11, 25]. Isarida and Isarida [23] complemented previous reviews with their analysis of context-dependent memory effects of simple and local contexts (e.g. background colors, odors, music). For background colors, they conclude that simple-context-dependent recall is determined by an item-by-item change in background and the effect disappears, when only one common background is used, or with only five or more successive presentations of the same background [23, 42, 43]. A possible cause for the disappearance of an effect with one common background is cue overload. Cue overload refers to a predominance of a context that is paired with multiple items, thereby reducing its meaningfulness for each individual item [2]. According to this proposal and following the design of previous studies [23, 41], contexts in our studies were paired with twelve items, which is thought to be small enough to avoid cue overload [23, 42]. Moreover, Fernandez and Glenberg [15] found that increasing the number of presentations of to-be-learned items reduced the effect of context on recall. Further, they assumed that items which are easier to remember are more likely to be influenced by contextual changes. In line with these suggestions, we chose familiar nouns as learning items and encoding comprised of only one presentation of each word. Although the number of to-be-learned words per context seems small (n = 12), this should not influence the effect of context reinstatement on memory performance, as Smith and Vela [2] pointed out in their meta-analysis that the number of learning items was not a predictor of the effect. As such we are confident that our overall design was well suited to detect context-dependent memory effects.

### Simple visual context reinstatement does not facilitate recall

Simple visual contexts did not affect recall performance as shown in Study 1 (Exp. 1.1). This experiment was carried out as a close replication of Isarida and Isarida (Experiment 1 in [23]), maintaining every critical methodological aspect of the original study. Various discriminating factors, such as cue overload, difficulty of the stimuli and number of subjects were closely adhered to. Cautiously, Isarida and Isarida (p. 1623 in [23]) wrote about their findings that “the reliability of the present results needs to be confirmed by replication”. Although an internal replication of their finding was successful (Experiment 2 in [23]), we failed to confirm their findings (Study 1). On the basis of our results, we cannot conclude whether concealed methodological differences caused these opposing results, or whether the original findings represent a Type I error and that a simple background color change is not sufficient to produce reliable context-dependent memory effects on recall. Further investigations are needed to clarify this uncertainty.

### Richness of visual cues does not facilitate contextual memory effect

While some studies have found effects of context reinstatement with simple visual cues [23, 44, 45], others argue that a significant effect on memory recall is more likely to be evoked by richer contexts with more information content. Murnane and colleagues demonstrated in a series of experiments that rich visual contexts, such as photographs with meaningful content, are more likely to be memorized alongside a learning item compared to simple background color changes [18–21]. Other studies investigating the salience of context tested memory effects via a recognition task reported better context-dependent discrimination between correct recognition and false alarms (e.g. [21, 46]). Although richer visual contexts, such as videos [41] or virtual environments [47] have been shown to produce context effects on recall memory, to our knowledge, no study that used different types of experiments, varying in the degree of visual richness, has been carried out for this test type. However, it is important to note that in most cases, where context-dependent discrimination was found, participants were instructed to *make use* of the presented context during learning by forming associations [14, 21, 46]. Contextual information that is explicitly learned and associated with the stimulus is more likely to serve as a memory aid during test [48]. In our studies, participants were not instructed to actively use contextual cues as a memory aid [25]. Incidental information derived from background contexts has been shown to be stored in memory and facilitate the retrieval process [11, 25, 49]. Although an instructed building of associations between learning item and its surrounding would naturally lead to a higher weighting of contextual information in memory recall, a more casual handling of environmental cues seems more alike everyday learning situations. Across our studies, which all used intentional encoding, the results provide evidence that visual cues, even rich and salient ones, do not facilitate a contextual memory effect.

### Use of VR makes no difference regarding contextual memory effect

By comparing the impact of a local on-screen context and a virtually presented and fully immersive environment, we tested further the effect of reinstated local versus global contexts on memory. To our surprise, whether an environment that is presented on a computer screen, or in a fully surrounding virtual manner, had little effect regarding contextual binding of the to-be-learned words, which is in line with the lack of experimental evidence in support of a context-dependent memory effect as reported above.

We used virtual reality head-mounted displays in Studies 2 and 3 (Exp. 2.2, Exp. 3.2, Exp. 3.3), a technology that is increasingly used in cognitive and neuroscientific research because it allows for the creation of immersive, three-dimensional environments that can be fully controlled by the experimenter [50]. The level of immersion is determined by the number of sensory channels connected to the virtual environment and the quality of the perceived stimuli (for a review: [51]). In our experiments, sensory stimulation was limited to the visual system, arguably providing moderate immersiveness. However, the whole visual field was stimulated and used as context, leading to a wider and more salient modulation compared to on-screen presentations. While virtual reality environments have been successfully used in other experiments to modulate context-dependent behavior (e.g. showing a location updating effect: [47], revealing context-specific conditioned fear memories: [52]), our studies revealed no such effect. Comparison of general memory performance revealed that the use of fully surrounding environments made no difference regarding memory performance compared to on-screen presentations.

Weak findings, as well as failures to find effects of context reinstatement on memory in previous studies have been explained with a suppression of the immediate environment. A suppression of contextual cues can be intentional, such as when participants avert their gaze or even close their eyes [53], however, our task does not allow for this strategy since all items are displayed visually. On the other hand, non-intentional suppression might occur when the activity or task being performed by the participant requires such concentration and attention that it overshadows the environmental cues in the learning environment or outshines any environmental cues if it occurs during retrieval. Participants’ individual mnemonic strategies could have led to such a requirement of concentration and therefore decreased attention towards the contexts [54], however, we tried to reduce the effect of overshadowing by using simple and highly familiar words. Thus, together with our results discussed above, our study revealed that displaying more salient environmental cues–either in a richer context or by stimulating more parts of the visual field–did not provide sufficient support to significantly enhance the participants’ memory recall [55].

### Neural reinstatement via visual flickering stimuli does not consistently evoke context dependent memory effects

While fully surrounding environments and on-screen presentations of colors or landscape pictures were consistently unable to evoke context-dependent effects on memory in our studies, flickering background screens (Exp. 2.3 and Exp. 3.3) showed varying results. The visual presentation of flickering stimuli (here: gray and white flickering of a computer screen) are known to reliably elicit steady-state visually evoked potentials (SSVEP’s) in visual areas of the brain [33]. Although we did not measure the electrophysiological product of this visual stimulation, decades of research support our assumption of frequency-dependent evoked responses according to our stimulation frequencies (for reviews: [33, 34, 35]). Steady-state responses are used in cognitive (e.g. working memory) and clinical neuroscience (e.g. epilepsy), in brain-computer interfaces (BCI’s) and most recently to depict reinstatement of oscillatory brain activity during successful retrieval of memorized information [28]. Neurophysiological evidence for neural reinstatement in memory tasks has emerged from studies, showing that certain brain activity from an encoding situation re-occurs during the successful retrieval of learned information [27–31]. Inspired by a recent study that was able to show an improvement of memory performance when oscillatory brain activity was experimentally modulated during the encoding of words and subsequently reinstated during retrieval [32], we modulated and reinstated neural oscillations in visual areas with the aim of evoking a context-dependent memory effect. As such, we aimed for a causal modification of memory performance by experimentally modulating oscillatory brain activity via external stimulation during encoding and recall. While previous studies used recognition tasks to show effects of neural reinstatement on memory performance [28, 32], we aimed for the same effect in a free recall task because previous studies showed that neural oscillatory patterns reoccur during memory search in free recall tasks [56–58]. In Experiment 2.3 (flickering stimuli on the screen) we were able to show improved memory performance of words with a reinstated neural context compared to words with no reinstatement. However, in Experiment 3.3 (flickering environment) we could not replicate this result when we used a HMD to present the visual flickering over the whole visual field. This design was chosen, firstly, to increase the possible influence of the visual context and secondly, to make an additional comparison of the general influence of the use of an HMD on memory performance. With an increased proportion of the visual field being stimulated, we would argue that steady-state responses would be induced in a greater area of the visual cortex and therefore lead to a stronger contextual trace alongside the learned items. Thus, to assume that steady-state responses from an on-screen presentation and HMD presentation would critically differ, seems unlikely. Studies have shown that reliable phase-locked responses can be evoked in the range from 1 Hz to 100 Hz when the whole visual field is presented with a flickering visual stimulation [59–61]. Additionally, general memory performance did not differ between both oscillatory reinstatement experiments. Note also that statistics of Experiment 2.3 revealed only anecdotal evidence in favor of a context dependent memory effect. Moreover, a sequential analysis of pooled data from Experiments 2.3 and 3.3 reveal that with an increasing sample size, evidence against the context-dependent memory hypothesis increases. However, this analysis might not have revealed yet a robust estimate of the true effect suggesting that either H1 and H0 are very similar, that there might be large inter-individual differences or that certain experimental parameters strongly influence the behavioral results. Together, these analyses suggest that flickering stimuli have only a small or very small effect on context-dependent memory recall at the group level. Future studies might investigate increased sample sizes and electrophysiological measures to uncover possible neural effects beyond behavioral performance.

### General memory performance does not differ between different visual contexts

Although the use of a HMD did not affect context-dependent memory, Experiment 2.2 revealed significantly better general memory performance compared to the on-screen experiments of Study 2 (Exp. 2.1 and Exp. 2.3). However, as suspected, this effect could be explained with a longer encoding period for each learning item and disappeared when the encoding duration was better controlled in Study 3. This finding is in line with previous research showing that the level of immersiveness, or the individual’s feeling of presence, is not associated with memory performance [62]. For example, Mania and Chalmers [63] compared three types of lecturing (real classroom, virtual classroom and auditory recording) and found no correlation between declared presence and memory performance. While only few studies were able to find a beneficial impact of an increased level of immersiveness on task behavior, the use of virtual reality devices have been shown to evoke reliable emotional responses [62]. For instance, various anxieties and phobias (e.g. acrophobia, arachnophobia, claustrophobia) can be elicited and treated with virtual reality devices (for a review: [64]).

### Limitations of our studies

Our current results challenge the view that reinstatement of visual context cues can influence memory recall. However, it is important to note how “reinstatement”, “context” and “memory” have been operationalized in our study. More specifically, our experiments show that under the chosen set of conditions context-dependent memory is more likely to be a myth than beneficial reality. Nevertheless, we acknowledge that other studies have found effects of visual context reinstatement on memory by using different methodological approaches (e.g. participants are instructed to pay attention to context: [41, 65]). In their recently published book Humphreys and Chalmers [66] discuss how context is used to control memory access and its effects on recognition, and summarize various studies showing that contextual information is stored alongside to-be-learned items. Note however, that we limit our conclusion to free recall memory tasks and our results likely do not generalize to other forms of memory retrieval (e.g. recognition). Another important distinction to previous studies is the use of a short consolidation time between encoding and recall. As discussed earlier, we chose our study design because it has been confirmed to evoke context-dependent effects on memory performance. However, other studies used longer retention intervals and some even modulated memory performance by experimentally interfering with memory consolidation (e.g. stimulus cueing during sleep: [67, 68]). Participants in our experiments executed a short distraction task, mainly to avoid serial-position effects [69]. Indeed, Smith and Vela [2] found in their meta-analysis that longer retention intervals (1 day to 1 week) increased the effect size of contextual reinstatement on memory. With the goal of replicating previous findings [23] we kept the consolidation duration short, although this might have diminished the contextual influence. Further, we only investigated the influence of context cues in the visual modality. Thus our results do not readily generalize to multimodal contexts (as used in [11]) or even other modalities like odor or sound. As discussed above, we did not encourage our participants to form active associations between the context cue and the to-be-remembered words. However, it has been shown that intentional encoding paired with incidental background contextual information is a suitable and often used paradigm for context-dependent memory studies investigating the influence of environmental contexts on memory performance. Moreover, we only controlled for the “external context” while the internal state of the participant was not experimentally modulated (except arguably neuronal oscillations in Exp. 2.3 and 3.3). However, also the internal state can represent a context relevant for memory recall (for a review on mood and state-dependent memory: [70]). Finally, we specifically tested the declarative memory domain. However, it is very likely that, for example, fear memories exhibit a much higher context sensitivity as shown in various studies focusing on fear conditioning in humans (e.g. [71, 72, 73]).

## General conclusion

In consideration of all seven experiments, we have to conclude that visual contextual information does not affect memory performance when reinstated during free recall. This conclusion, although surprisingly consistent across various levels of visual contexts, is in line with previous studies showing only weak or no effects of context-dependent memory [12–15, 25] and echoes the seminal study of Godden and Baddeley (p. 325 in [11]), stating that “the evidence for context-dependent memory is therefore far from convincing”. However, caution is advised when generalizing our findings to other context or memory modalities or to everyday learning. The process of memorizing information in everyday situations represents an overlap of coherent events [15], where context is usually closely related to the information and consists of a variety of sensory, cognitive and emotional features. We showed that selectively changing visual cues independent from the to-be-learned information is not sufficient to elicit context-dependent memory which challenges current textbook knowledge and limits the applicability of this strategy in real-world settings.

## Supporting information

S1 Table Order effects in Studies 2 and 3. Comparing context reinstatement effect regarding the order of the experiments in Studies 2 and 3. One-sided paired t-tests were used to compare reinstated and non-reinstated recall performance in the first, second and third experiments. P-values marked with an asterisk were derived from Wilcoxon signed-rank test because a test of normality (Shapiro-Wilk) revealed a significant deviation of a normal distribution.
(PDF)

S2 Table Comparison between first and second half of recall. Two-sided paired t-tests were used to compare reinstated and non-reinstated recall performance in the first half of the recalled words, as well as in the second half of the recalled words.
(PDF)
