# Supplementary material for: Reinstating verbal memories with virtual contexts: Myth or reality?
Source: PLoS One. 2019 Mar 29;14(3):e0214540. doi: 10.1371/journal.pone.0214540 (PMC6440692; doi:10.1371/journal.pone.0214540)
Supplement: S1 Table — Comparing context reinstatement effect regarding the order of the experiments in Studies 2 and 3. One-sided paired t-tests were used to compare reinstated and non-reinstated recall performance in the first, second and third experiments. P-values marked with an asterisk were derived from Wilcoxon signed-rank test because a test of normality (Shapiro-Wilk) revealed a significant deviation of a normal distribution. (PDF) [file pone.0214540.s001.pdf]

Study	Order of Experiment	Condition	Number of Samples	Mean Recalled Words	Standard Deviation	p-value
2	1	Reinstated	40	5.775	2.348	**0.356**
		Not-Reinstated	40	5.650	2.190	
	2	Reinstated	40	6.025	2.315	**0.133**
		Not-Reinstated	40	5.575	2.630	
	3	Reinstated	40	5.700	2.989	**0.580**
		Not-Reinstated	40	5.775	2.646	
3	1	Reinstated	40	5.850	2.434	**0.416**
		Not-Reinstated	40	5.775	2.281	
	2	Reinstated	40	6.000	2.501	**0.555 ***
		Not-Reinstated	40	6.025	3.125	
	3	Reinstated	40	5.875	2.839	**0.417**
		Not-Reinstated	40	5.800	2.633	
