# Supplementary material for: Reinstating verbal memories with virtual contexts: Myth or reality?
Source: PLoS One. 2019 Mar 29;14(3):e0214540. doi: 10.1371/journal.pone.0214540 (PMC6440692; doi:10.1371/journal.pone.0214540)
Supplement: S2 Table — Two-sided paired t-tests were used to compare reinstated and non-reinstated recall performance in the first half of the recalled words, as well as in the second half of the recalled words. (PDF) [file pone.0214540.s002.pdf]

Study	Experiment	1st/ 2nd Half	Condition	Number of Samples	Mean Recalled Words	Standard Deviation	p-value
1	1	1st	Reinstated	40	2.775	1.423	**0.940**
			Not-Reinstated	40	2.750	1.410	
		2nd	Reinstated	40	2.575	1.357	**0.239**
			Not-Reinstated	40	2.950	1.395	
2	2.1	1st	Reinstated	40	2.725	1.519	**0.320**
			Not-Reinstated	40	3.025	1.271	
		2nd	Reinstated	40	2.750	1.056	**0.372**
			Not-Reinstated	40	3.000	1.601	
	2.2	1st	Reinstated	40	3.125	1.604	**0.509**
			Not-Reinstated	40	3.375	1.690	
		2nd	Reinstated	40	3.550	1.679	**0.078**
			Not-Reinstated	40	2.950	1.413	
	2.3	1st	Reinstated	40	3.175	1.678	**0.090**
			Not-Reinstated	40	2.575	1.466	
		2nd	Reinstated	40	3.000	1.536	**0.447**
			Not-Reinstated	40	2.750	1.532	
3	3.1	1st	Reinstated	40	3.375	1.564	**0.308**
			Not-Reinstated	40	3.000	1.710	
		2nd	Reinstated	40	3.100	1.661	**0.662**
			Not-Reinstated	40	3.275	1.768	
	3.2	1st	Reinstated	40	3.325	1.509	**0.293**
			Not-Reinstated	40	2.950	1.724	
		2nd	Reinstated	40	3.125	1.842	**0.953**
			Not-Reinstated	40	3.150	1.733	
	3.3	1st	Reinstated	40	2.975	1.702	**0.462**
			Not-Reinstated	40	2.750	1.354	
		2nd	Reinstated	40	2.725	1.450	**0.404**
			Not-Reinstated	40	3.000	1.710	
